# Barriers to quality oral health care among elderly (Geriatric) patients in Oyo State

**DOI:** 10.4314/ahs.v26i2.18

**Published:** 2026-06

**Authors:** Idowu Aderemi Akinpelu, Abimbola Olubunmi Balogun, Akinyele Olumuyiwa Adisa, Juliana Obontu Taiwo

**Affiliations:** 1 Osun State Hospitals Management Board; 2 Department of Family Dentistry, University College Hospital, Ibadan, Oyo state, Nigeria; 3 Department of oral Pathology, University college Hospital, Ibadan

**Keywords:** barriers, elderly, oral care

## Abstract

**Background:**

The treatment needs of elderly people can be complex because of reduced mobility, compromised physical and mental states amongst others. These complexities can be pronounced in those with systemic diseases and those on medications that compound oral conditions e.g. Xerostomia. This makes oral hygiene and treatment more difficult. Despite their increased need, the uptake of dental care by elderly people is characteristically poor and unmet needs may be high^6^. Barriers to the uptake of care include a lack of perceived need, concerns about availability, cost and fear of cost, fear of treatment and anxiety about Dentist characteristics

**Materials and Method:**

This cross-sectional study sampled 500 elderly patients, aged 65 to 91, attending clinics in selected healthcare centers in Oyo State, Nigeria. A multistage sampling technique was used, and data was gathered through structured interviewer-administered questionnaires and a modified WHO clinical assessment form to assess oral health status. Data was analyzed using SPSS version 23, with summary statistics like frequency tables and means. Chi-square tests were employed to examine associations between variables, with significance set at P < 0.05.

Data collection was conducted daily, with verification and supervision to ensure accuracy. Occasional re-examination of random patients was done to maintain consistency among examiners.

**Result:**

The results showed a mean age of 74 ± 10 years, with 51% female participants. A mean DMFT score of 5 and a CPITN score of 2 indicated significant dental care needs. However, only 8.6% of the elderly participants had accessed dental care in the past 12 months. Financial constraints, fear of pain, and anxiety about dental instruments were some of the major factors preventing the elderly from seeking care.

**Conclusion:**

The study concluded that despite available public-sector dental services, financial and personal barriers contributed to unmet oral health needs among the elderly

## Introduction

Ageing can be chronological, biological or psychological^1^. Chronological age is a person's age in years. This has limited significance when talking about health^1^. Biological age simply refers to changes in the body which certainly occur as we age. The first sign may be in our appearance which includes gray hair, receding of hairlines, few wrinkles, weight gain, declined organ functions, impaired vision and hearing^2^. Psychological age is measured by how people act and feel. Decline in learning and memory usually begin after people reach their 70s and various disorders can set in, including loss of memory, mental or emotional disorders^3^.

Nowadays, people are not only living longer but also retaining their natural teeth for longer into old age^5^. Exposure of vulnerable root surfaces to the oral environment due to gum recession potentiates the decay process. Therefore older people are still at risk of developing root caries, gum disease, tooth wear and also oral cancer^5^.

Oral health care must be accessible to every segment of the population so as to bridge the gap between the need for care and the amount of care being sought. In order to achieve this, the barriers preventing elderly people from accessing the oral health care services must be put into consideration^6^. Despite their increased need, the uptake of dental care by elderly people is characteristically poor and unmet needs may be high^6^. A previous study among an American population demonstrated that barriers to the uptake of care include a lack of perceived need, concerns about availability, cost and fear of cost, fear of treatment and anxiety about Dentist characteristics^6^. However, owing to the current advances in oral health delivery and the increasing oral health awareness in our environment there's a need to investigate the prevailing barriers to oral heath among the elderly population in our environment.

The purpose of this study is to identify the barriers to quality oral health care among elderly (Geriatric) patients in Oyo-state and also to determine factors that will improve oral health service utilization. This is necessary before an effective dental public health programme can be instituted.

### Aim of the study

To identify barriers to quality oral health care among elderly (Geriatric) patients in Oyo State.

## Materials and method

This study was a cross-sectional study carried out amongst elderly patients attending 8 selected clinics (4 in U.C.H, Ibadan, 2 in Primary health care centres and 2 in state hospitals) in Oyo State. Elderly patients aged 65 years and above attending four selected clinics in UCH, Ibadan, two selected Primary health care centres, and two selected State hospitals in Oyo State.

Samples were collected from primary, secondary and tertiary centres. Using non-proportional stratified sampling technique, four departments (Geriatric, General Outpatients, Dental and Cardiology) were selected from the tertiary institution [University College Hospital]. 50 elderly patients were selected from each department by ballot. Using multistage sampling technique, from a list of Primary health care centres in Oyo State, two centres were selected using table of random numbers. Also from a list of secondary health care centres, two centres were selected using table of random numbers. Elderly patients were recruited into this study from these centres (Appendix H). One hundred and fifty patients were selected from primary health care centres and one hundred and fifty patients from secondary health care centres using ballot method.

Only elderly who fell between the ages of 65 years and above and who have at least 20 functioning teeth were allowed to partake in the study. Ethical approval for this study was obtained from the joint UI/UCH ethical review committee. Signed written informed consent was obtained from the patients.

The instrument of measurement consisted of a structured interviewer-administered questionnaire, a modified WHO clinical assessment form, CPITN Probe, Williams Probe, Dental Probe, Dental Mirror and Wooden Spatula.

The questionnaire had four sections. Section A was about Biodata of the patient. This includes Age, Gender, Marital status, Educational status, Religion, Occupation, Location and Ethnic group. Section B is about Knowledge and attitude towards oral health. This includes knowledge on dental clinics and dental treatment, previous dental consultation, how frequently they attend clinic and reasons for attendance. Section C is about Oral health care practices. This includes how often they brush their teeth on daily basis, what they use in cleaning their teeth, what they use in cleaning in-between their teeth, avoidance of carious causing diet and consumption, and any form of habit they have. Section D represents questions on the Barriers to Oral health care. The Assessment form is a modified WHO assessment form which consists of short biodata, serial code, CPITN index, caries index, and any other oral pathology.

Williams Probe was used to measure clinical attachment loss. This was measured from the cemento-enamel junction (CEJ) to the base of the pocket or sulcular depth. It is flat and evenly graduated (1mm) from 1 to 10. 4mm and 6mm are absent for ease of identification of the markings. 4 and 6 mm were chosen because 4mm is the upper limit of moderate Periodontitis and 6mm is considered advanced (> 5mm) Periodontitis^1^,^16^.

A preliminary study using 20 people to ascertain diagnostic criteria was carried out. The preliminary study was used to assess the adequacy and reliability of data collection methods and necessary modification was made to the questionnaire. The two examiners examined the same 20 people on two different occasions and the results were compared. Then intra- and extra-examiner variability was calculated using Cohen's Kappa with level of significance set at p>0.05.

The elderly patients attending the clinics were first interviewed using the questionnaire by a trained house officer after which he/she was examined by a calibrated resident doctor using a mouth mirror, a blunt dental probe of diameter 0.5mm and a CPITN (Community Periodontal Index of Treatment Need) probe (single banded) under fluorescent light while the recorder clerk recorded all the findings of the examination on the assessment form. Caries was assessed using the DMFT while periodontal disease was assessed using a CPITN index.

Data collection and verification were done on daily basis while the computer data entry was carried out after the required number of elderly patients was seen. The data gathering process was strictly supervised by the investigator and occasionally 20 elderly patients were re-examined at random to ensure consistency of the examiners. The data was then analyzed on the computer as well as manually.

Data was first collated, and then analyzed using the statistical package for social sciences (SPSS Inc. Chicago) version 23. Frequency distribution of variables such as age, gender, marital status, barriers to dental care was then generated. Data was presented using summary statistics such as frequency tables, charts, multivariate analysis, means and standard deviation. Chi-square test was used to test association between categorical variables; with level of significance set at p< 0.05.

Questions on barriers to quality oral health care were dichotomized; strongly disagree, disagree and ‘I don't know’ were regarded as negative responses while strongly agree and agree were considered positive responses respectively.

## Result

The age distribution of respondents who participated in this study ranged from 65 to 91 years. The mean age was 74 ± 10 years. The gender distribution was 49.0% male and 51.0% female. Additional socio-demographic variables considered included gender, ethnicity, religion, education level, occupation, and geographic location, as detailed in Table 1. The majority of respondents (36.6%) were within the 65–69-year age range

**Table 1:** 

Socio Demography	No. of Participants	Percentage (%)
**Age**		
65-69years	183	36.6
70-74years	102	20.4
75-79years	103	20.6
80 years and above	112	22.4
Total	500	100.0
**Gender**		
Male	245	49.0
Female	255	51.0
Total	500	100.0
**Tribe/Ethnicity**		
Yoruba	402	80.4
Ibo	67	13.4
Hausa	31	6.2
Total	500	100.0
**Religion**		
Christianity	282	56.4
Muslim	193	38.6
Others	20	4.0
Undecided	5	1.0
Total	500	100
**Education**		
Primary	42	8.4
Secondary	102	20.4
Post-secondary	123	24.6
Tertiary	166	33.2
no formal education	67	13.4
Total	500	100.0
**Marital status**		
Married	277	55.4
Single	4	.8
Divorced	43	8.6
Separated	63	12.6
Widow/widower	113	22.6
Total	500	100.0
**Occupation**		
Artisan	52	10.4
Retiree	172	34.4
Trading	276	55.2

## Discussion

The characteristics of the elderly make them a unique population as manifestation of ill health in them is distinct from the rest of the population^16^. The mean age was 74years + 10years and most of the participants were between 65-69years old. We had more female (51%) participants than males and majority were from Yoruba tribe. In term of religion, Christianity (56.4%) predominates among the participants and most have tertiary education (33.2%). The number of married people predominates with a percentage of 55.4%. Majority of the participants are traders (55.2%) and many are indigenes of Ibadan. Like in most other similar epidemiological studies^10^, ^1^,^17^, more female respondents (51.0%) were involved in this study. In relation to oral health seeking behaviour, many of the patients in this study visit the dental clinic only when there was episode of pain. This is not a new finding in this environment as visits to dental care facilities are said to be mostly for symptomatic reasons rather than for preventive measures^18^.

It was discovered that many people did not go to dental clinic frequently within a space of six months. The percentage of the participants who visit dental clinic once in less than six months was 14.6% while 8.6% visit dental clinic once in more than six months but less than a year. More so, about 44.8% of the respondents do not visit dental clinic in more than 1year. This is consistent with study of Taiwo et al 2004^18^.

It was discovered that the main reason why most of the participants (45.8%) went to the dental clinic was mainly for extraction, some went for scaling and polishing (14.0%) while minority (9%) went for check-up. About 31.2% of the participants did not respond to the question. Cost and fear of cost constituted the barrier with highest ranking among the respondents in this study (62.0%). The expensive nature of dental care has consistently remained a highly rated barrier to oral health utilization worldwide especially in developing countries like Nigeria with unstable resources and lack of robust medical insurance^20^. Likewise in Varenne and Guay studies, high cost of dental treatment was considered the strongest barrier to oral health care^10^,^18^.

Fear of pain from treatment ranked next to cost of treatment in this study. Avoidance of dental care due to fear is a well-recognized phenomenon in oral health care surveillance. Associations have also been found between dental fear and less frequent dental consultation and a consistent finding is that females have a greater prevalence of fear than the males^21^. This is consistent with the findings of Fitzpatrick et al 2004^25^.

Feeling insecure while undergoing dental treatment as ranked by respondents may be attributed to fear of contracting infection^22^. In our environment, HIV cross-infectivity especially from blood transfusion exists in the literature and this kind of report can make elites nervous when they seek oral healthcare^22^.

Poor attitude to work by dental staff, noise from dental instrument, transportation, problem and busy schedule constituted weakest barriers in this study. Inability to gain access to oral health facilities as a result of poor transport system, living in rural areas, and poor systemic health have been described in dental literature^10^.

Periodontal evaluation using the CPITN revealed over half (62.2%) of the respondents experienced periodontal diseases. The presence of calculus (33.8%) and shallow pocketing (30.4%) were predominant features. About 11.2% of the participants have deep pockets. This was comparable to results of the periodontal status of the Black and Indian population in the National oral health survey^23^.

The mean DMFT score for ages 65- 69years was 3.29, ages 70-79years was 1.57 and 80years and above have 5.04 mean DMFT. The M component is the cause of increasing mean DMFT with age. This is consistent with findings of Taiwo et al 2007^24^.

The younger elderly (65-69years) have healthier periodontium than the rest of the elderly whilst the 80years old and above have the worst periodontal health (P=0.04) and this was statistically significant. Periodontal health deteriorates with age. The age group between 70 and 74 years had more accumulation of calculus than the younger age group. This is not farfetched from the fact that with increasing age there is loss of manual dexterity due to arthritis and reduced muscle tone. This is consistent with the findings of Taiwo et al 2012^24^.

## Conclusion

The majority of the elderly were in the group of younger elderly i.e 65-69years. The major barriers were fear of pain from treatment (45.2%), fear of injection (59.8%) and cost (62%) as participants strongly agree and agree respectively, indicating need for improved health education for elderly on dental charges and care pathways in Oyo State. Barriers had a negative impact on oral health status of the elderly. There was a strong association between age and barriers and this shows that age is one of the determinants in considering barriers to dental or oral care.

## Figures and Tables

**Table 2:** Dental Clinic Attendance

Dental clinic attendance		No. of participants	Percentage (%)
	Less than 6 months	73	14.6
	More than 6 months but less than a year	43	8.6
	More than 1 year	224	44.8
	No response	160	32.0

	Total	500	100.0

(*Majority have not attended the dental clinic in the last one year)*

**Figure 1:**
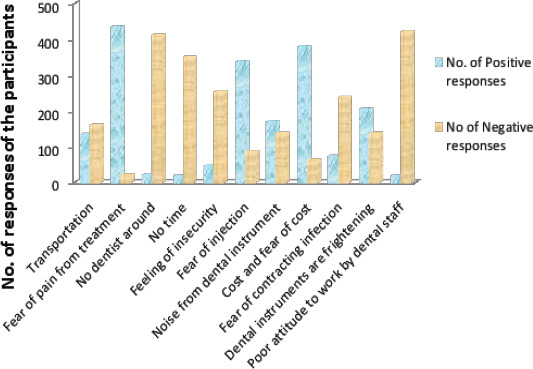
Barriersto dental and oral care

(Fear of pain and cost were the major barriers observed among the subjects)

**Table 3:** Dental caries experience among the study population

Age	No. of patients	Mean DMFT	Std. Deviation DMFT
65 to 69years	183	3.29	1.827
70 to 74years	103	0.79	0.68
75 to 79years	102	0.78	0.66
80years and above	112	5.04	2.084
Total	500	2.98	2.172

*(Highest prevalence of caries was observed among subjects aged 80 years and above)*

**Figure 2:**
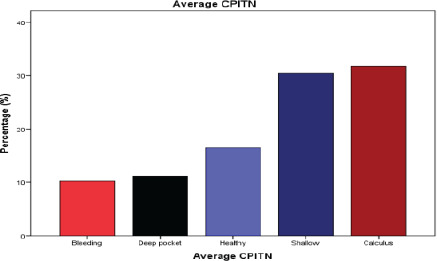
periodontal status of the study population

(Many of the subjects had shallow pockets and calculus accumulation)

**Table 4:** Impact of barriers on oral health status (CPITN and DMFT)

Source	Dependent Variable	Type III Sum of Squares	df	Mean Square	F	Sig.
	DMFT	478.148	18	26.564	6.791	.001
Barriers	CPITN	26989.44	18	1499.41	14.832	.001

*(the identified barriers had statistically significant impact on the caries experience and CPITN score p=0.001)*

## References

[1] Collins dictionary of sociology (2003). Definition of elderly. Charpter One.

[2] Hooyman NR, Kiyak HA (2011). Social gerontology: A multidisciplinary perspective.

[3] Centres for Disease Control and Prevention and the Merck Company Foundation (2007). The state of aging and health in America.

[4] Mortelmans E, Berghmans J, Justens M (2008). Quality dental care: a complex subject. Rev Belge Med Dent.

[5] Borreani E, Wright D, Scrambler S (2010). Informing the debate on oral healthcare for older people. Gerodontology.

[6] Guay AH (2004). Access to dental care. Solving the problem for underserved populations. J Am Dent Assoc.

[7] National working Group for older people (2005). Meeting the challenges of oral health for older people: a strategic review. Gerodontology.

[8] Brodeur JM, Demers M, Simard P, Kandelman D (1988). Need Perception as a major determinant of dental healthcare utilization among the elderly. Gerodontics.

[9] Kay DW (1989). Ageing of the population: Measuring the need for care. Ageing.

[10] Guay AH (2004). Access to dental care. Solving the problem for underserved populations. J Am Dent Assoc.

[11] Taylor GW, Borgnakke WS (2008). Periodental disease: association with diabetes, glycemic control and complications. Oral Diseases.

[12] Thane P (1978). The muddled history of retiring at 60 and 65. New society.

[13] Marybeth W (2001). Personnel correspondence.

[14] Jary D, Jary J (2003). Collins Dictionary of Sociology. Definition of elderly.

[15] Masoro EJ, Boron WF (2012). “The Physiology of Aging”. Medical Physiology.

[16] World Health Organizations (1998). Scientific Group on the Epidemiology of Ageing: The use of Epidemiology in the study of the elderly. WHO Tech Rep Ser 706.

[17] Zizza CA, Ellison KJ, Wernette CM (2009). Total water intakes of community-living middle–old and oldest-old adults. Journals of Gerodontology.

[18] Taiwo JO, Noah M (2004). Pattern of dental clinic attendance of registered diabetic patients in Ibadan. Journal of Medicine and Biomedical Research.

[19] Togunu-Bickersteth F (1988). Perception of old age among Yoruba aged. Journal of Comparative Family studies.

[20] Puska P, Pietenren P, Uusitalo U (2002). Influencing public nutrition for non-communicable disease prevention. Public Health Nutr.

[21] Ramsay SE, Wannamethee PH, Whincup RG (2015). Burden of poor oral health in older age: Findings from population-based study of older British men. British Medical Journal (BMJ).

[22] Rise J, Hollund U (1990). Prediction of sugar behaviour. Community dental health.

[23] Ronnie L (1989). The scientific basis of dental health education a policy document.

[24] Taiwo JO, Ibiyemi O, Bankole O (2012). Oral Health Attitudes and Practices of the Elderly People in South East Local Government Area (SELGA) In Ibadan. Journal of Biology, Agriculture and Healthcare.

